# Effect of Telmisartan in the Oxidative Stress Components Induced by Ischemia Reperfusion in Rats

**DOI:** 10.1155/2019/1302985

**Published:** 2019-07-02

**Authors:** Simón Quetzalcoatl Rodríguez-Lara, Walter Angel Trujillo-Rangel, Araceli Castillo-Romero, Sylvia Elena Totsuka-Sutto, Teresa Arcelia Garcia-Cobián, Ernesto German Cardona-Muñoz, Alejandra Guillermina Miranda-Díaz, Ernesto Javier Ramírez-Lizardo, Leonel García-Benavides

**Affiliations:** ^1^International Program, Instituto de Ciencias Biomédicas, Universidad Autónoma de Guadalajara, A.C. Av. Montevideo, esq. Av. Acueducto, Col. Prados Providencia, Guadalajara, Jalisco, C.P. 44670, Mexico; ^2^Instituto de Terapéutica Experimental y Clínica, Departamento de Fisiología, CUCS, Universidad de Guadalajara, Calle Sierra Mojada 950, Colonia Independencia, CP 44340 Guadalajara, Jalisco, Mexico; ^3^Departamento de Microbiología y Patología, CUCS, Universidad de Guadalajara, Calle Sierra Mojada 950, Colonia Independencia, CP 44340 Guadalajara, Jalisco, Mexico; ^4^Departamento de Ciencias Biomédicas, CUTonala, Universidad de Guadalajara, Avenida Nuevo Periférico No. 555, Ejido San José Tateposco, CP 45425 Tonalá, Jalisco, Mexico

## Abstract

The therapeutic effects of telmisartan, an angiotensin II receptor antagonist and a peroxisome proliferator-activated receptor-*γ* (PPAR-*γ*) agonist, have been demonstrated in several disorders. It has antioxidant and immune response modulator properties and has shown promising results in the treatment of an ischemia/reperfusion (I/R) lesion. In this study, a skeletal muscle (right gastrocnemius muscle) I/R lesion was induced in rats and different reperfusion times (1 h, 24 h, 72 h, 7-day, and 14-day subgroups) were assessed. Furthermore, levels of oxidative markers such as enzymatic scavengers (catalase (CAT) and superoxide dismutase (SOD)) and metabolites (nitrates and 8-oxo-deoxyguanosine) were determined. The degree of tissue injury (total lesioned fibers and inflammatory cell count) was also evaluated. We observed an increase in CAT and SOD expression levels under telmisartan treatment, with a decrease in injury and oxidative biomarker levels in the 72 h, 7-day, and 14-day subgroups. Telmisartan reduced oxidative stress and decreased the damage of the I/R lesion.

## 1. Introduction

Ischemia reperfusion (I/R) is a phenomenon that occurs after the occlusion of arterial blood flow to a specific organ or tissue. The ischemia causes an imbalance in metabolic substrate levels on the exposed cells; depending on the hypoxia duration, several mechanisms, which can help cell survival or induce further damage, are activated [1–3]. Reperfusion is the restoration of the blood flow and reoxygenation of the affected tissues. It is related to an exacerbation of the initial lesion and followed by several physiopathological mechanisms in the affected cells, such as increments of cations in cytosol, formation of reactive oxygen species and nitrogen species, disruption of the signaling redox (oxidative stress (OS)), mitochondrial lesion, transcriptional reprograming, endothelial lesion, no-reflow phenomenon, immunity-mediated lesion, apoptosis, autophagy, and necrosis. From all these processes, the pathological changes in the affected tissue can be divided into acute (first 72 h) and chronic (15–90 days) at onset, which are determined by factors, such as the time of ischemia and the response to inflammation and oxidative stress [3, 4]. These two processes produce tissue damage, leading to the malfunction or dysfunction of the affected tissues by many mechanisms [3, 4]. The I/R phenomenon impact is most pronounced in patients with myocardial infarction or cerebral ischemia. Its repercussions can produce deleterious effects in the patients, and their prevalence affects the health care system [5–8]. Despite the impact of this phenomenon, no specific pharmacological approach has been established to decrease lesion development [9].

In the OS component of the I/R lesions, the role of enzymatic scavengers such as superoxide dismutase (SOD) and catalase (CAT) is crucial for cell survival [1–3], not only because they are the first line of defense against reactive oxygen radicals (ROS) and nitrogen (RNS) but also because their absence or malfunction leads to signaling redox disruption in the cell, inducing or increasing the I/R lesion. The I/R lesion formation process may be complex because of all the pathophysiological components, but the increase in enzymatic scavengers' expression, function, or activity may partially improve the affected tissue survival [10, 11]. Several authors have described that telmisartan, an angiotensin type 1 receptor antagonist and peroxisome proliferator-activated receptor-*γ* (PPAR-*γ*) receptor partial agonist, produces a beneficial effect on OS-induced damage in different pathological process (diabetes, dyslipidemia, hypertension, cancer, etc.) and has shown encouraging results even in the animal models of I/R lesions [12–22]. Telmisartan has been shown to affect the concentration and activity of some enzymatic scavengers (SOD, CAT, NADPH, etc.), but its mechanism of action and the impact on the extent of I/R lesion progression have not been well elucidated [12–22]. The effects of the PPAR-*γ* receptor activation have shown expression in many tissues, but its maximal expression has been shown in the adipocyte and immune cells. The pathways involved in the signaling effects are related to the activation of the extracellular signal-regulated kinase–mitogen-activated protein kinase (ERK-MAPK) cascade, which can induce activation and differentiation of macrophages, increasing the expression of CD36, stimulating recruitment activity, and inducing the survival of the cells under OS [23, 24].

We aimed to determine the effect of telmisartan on the expression of SOD and CAT and on the production of oxidative metabolites like nitrates (products of protein nitrosylation) and 8-oxo-deoxyguanosine (products of DNA oxidation). Furthermore, we evaluated the histological impact of telmisartan on I/R lesion-induced muscle tissue damage in the limbs of Wistar rats.

## 2. Material and Methods

### 2.1. Animals

Male Wistar rats, weighing 250–350 g, were housed in colony rooms with a 12/12 h light/dark cycle at 21–25°C and had free access to food and water. All animal experiments were performed in accordance with the Centro Universitario de Ciencias de la Salud of the Universidad de Guadalajara ethical committee regulations (register number: CEI/19/2015). All guidelines for laboratory animal care of the Mexican legislation were followed (NOM-062-ZOO-1999).

### 2.2. Pharmacological Intervention

Telmisartan was obtained in the form of tablets (from Boehringer Ingelheim) that were powdered and titled according to each rat's weight, using a sterile technique, diluted with injectable water, vortexed, and administered using an orogastric cannula. The drug was administered at single doses of 20 mg/kg/day, which was selected taking under consideration other studies [22], in the morning in the therapeutic intervention group, whereas injectable water was used as vehicle for the no therapeutic intervention group. The rats received telmisartan or injectable water daily for 7 days prior to the induction of an I/R lesion and following the induction of the lesion until the sacrifice of the animals at the specified time points.

### 2.3. Experimental Procedure to Induce Ischemia Reperfusion

All rats were anesthetized with an intraperitoneal injection of Zoletil 50 (tiletamine 125 mg/zolazepam 125 mg) at a dose of 40 mg/kg and ketamine at a dose of 80 mg/kg [25]. No additional anesthesia administration was necessary during the procedure.

The skin in the area of the procedure in the right inguinal fold was disinfected and shaved; a 1 cm incision was made and dissection was performed using a dulled technique until the vascular-nerve plexus. A tourniquet was placed on the entire plexus using prolene 3-0 to induce ischemia in the gastrocnemius muscle, and the incision was closed using silk 3-0. Ischemia was maintained for 6 h. Following this, the animals were anesthetized again; the incision was reopened; and for reperfusion, the tourniquet was removed, avoiding damage to the plexus, using a fine blade under a magnifying glass and direct visualization. After reperfusion, the animals were sacrificed under anesthesia; blood and gastrocnemius muscle samples were obtained for RNA extraction and histological analysis, as well as for SOD, CAT, and 8-oxo-deoxgyuanosine quantification using enzyme-linked immunosorbent assay (ELISA) and nitrite/nitrate quantification using colorimetric analysis.

### 2.4. Experimental Protocol

Overall, 50 rats were included in the experiment and were divided into two groups of 25 rats each. Group 1 received a therapeutic intervention (telmisartan), whereas group 2 received only injectable water.

**Figure 1:**
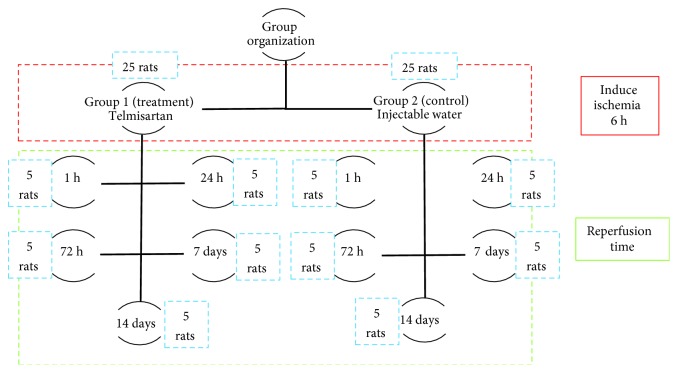
Group organization. Ischemia was induced for 6 h in all groups.

Both groups were subdivided into five subgroups (each comprising 5 rats) based on 1 h, 24 h, 72 h, 7 days, and 14 days of reperfusion (Figure 1).

### 2.5. Gene Expression

The muscle samples were stabilized using RNAlater from Thermo Fisher Scientific (Cat. AM7020) and frozen at −80°C until RNA extraction was performed. RNA extraction was performed from the muscle samples within 24 h following the sacrifice. The samples were cut in 50 to 100 mg pieces, using a sterile technique, and homogenized with TRIzol from Invitrogen (Cat. 15596026) and TissueRuptor from QIAGEN. RNA concentration and integrity were evaluated using the Eppendorf NanoDrop spectrophotometer. RNAs isolated from different samples had similar gel electrophoresis profiles. Total RNA yield from each tissue sample (50–100 mg) equaled at least 150 *μ*g and was sufficient for endpoint QRT-PCR analysis. cDNA synthesis was performed using Verso cDNA synthesis from the Thermo Fisher Scientific kit (Cat. AB1453A) in a volume of 20 *μ*L: cDNA synthesis 4 *μ*L, dNTP mix 2 *μ*L, RNA primer 1 *μ*L, RT enhancer 1 *μ*L, Verso enzyme mix 1 *μ*L, template RNA 5 *μ*g, and DEPC water up to 20 *μ*L.

The primer was designed using the sequences from the GenBank of the PubMed database and the program FastPCR 6.5 version. The primers had the following sequences: (1) for SOD-2, FW TTC AGG GAA GCC ATT CAG CAC CAT and RW ACT GTT ACC TTG TCA GGG A; (2) for CAT, FW TTG GCA GAG CCT GAA GTC ACC ACT and RW TGG TCA GGA CAT CGG GTT TCT G; and (3) for *β*-actin (constitutive), FW AGT ACA ACC TTC TTG CAG CTC and RW AGT CCT TCT GAC CCA TAC CC.

The gene expression levels were assessed using endpoint QRT-PCR. The amplification reactions were performed in a volume of 10 *μ*L using the Thermo Fisher Scientific DreamTaq Green PCR Master Mix (Cat. K1089BID). The procedure was performed using the DreamTaq Green PCR Master Mix 5 *μ*L, FW primer 0.5 *μ*L, RW primer 0.5 *μ*L, template 1 *μ*L, and injectable water 3 *μ*L. Tm-normalized hybridization conditions were as follows: initial denaturalization for 1 min at 95°C, 40 amplification cycles (30 s/95°C, 30 s/57°C, and 1 min/72°C), and extinction for 7 min at 72°C. Hybridization was performed in triplicates in a Thermo Scientific thermal cycler. Samples were run on a 1% agarose gel at 70 mV, 400 amp for 45 min in a Bio-Rad electrophoresis chamber and visualized in a 360 nm transilluminator; the obtained images were evaluated using the program “ImageJ” for all sample quantifications [26].

### 2.6. Biochemical Assay

SOD-2, CAT, and 8-oxo-dG residue protein levels were measured using sandwich ELISA kits (R&D Cat. DYC3419-5, MBS Cat. 701908, and Trevigen Cat. 4380-096-k, respectively). Nitrite concentration was determined using a colorimetric assay kit (Sigma cat. 23479-1KT-F). All experimental procedures were performed following the manufacturers' specifications. The Sinergy HT spectrophotometer was utilized for processing and the program GEN5 11 version for curve plots and calculations.

### 2.7. Histological Tissue Evaluation

The tissue samples were immersed in 4% paraformaldehyde and processed into 5 *μ*m sections from the proximal, medial, and distal areas of the right gastrocnemius muscle. They were subsequently processed in a paraffin bath and stained with hematoxylin and eosin. Microscopic evaluation was performed under an Axiostar plus microscope. Images were captured using the Carl Zeiss Axiocam, and measurements were performed using the AxioVision 8.1 program.

Cell and tissue damage was evaluated independently by three different pathologists, using full-frame counting, fourfold, and stratified techniques [27]. The lesioned muscle fibers were assessed individually, and inflammatory cells were accounted for.

## 3. Statistical Analysis

Data are expressed as mean ± standard error of the mean. Normality of the data was assessed using the Shapiro–Wilk test. For between-group comparisons, the Kruskal–Wallis test, followed by the Mann–Whitney *U* test, was used. *p* values < 0.05 were considered significant.

## 4. Results

### 4.1. Effect of Telmisartan on Gene Expression

Data quantification was performed using the ImageJ program by measuring optical densities (ODs) [28]. OD normalization for each sample was performed using a constitutive gene (*β*-actin). For gene expression comparisons, OD from the no treatment group was divided by that from the treatment group. The normalization procedure was performed by measuring the gel image of each gene in triplicates, averaging the values, and dividing the constitutive gene value by the corresponding gene sample value. For the comparison of samples, the normalized values of the expected gene were divided by the values of the gene of interest ((I/R)/(IR + Telm)). Data were normalized to 1 and plotted in arbitrary expression units (AEUs) for analysis using GraphPad Prism 6.1 [29].

**Figure 2:**
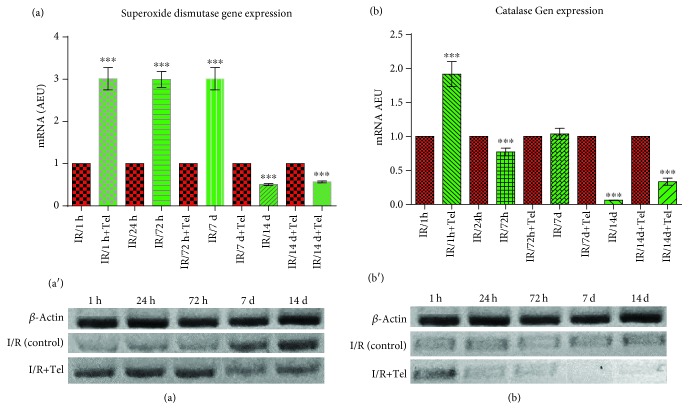
Gene expression of superoxide dismutase and catalase. (a) Comparison catalase gene expression between group 1 and group 2. (a′) Comparison of the agarose 1% gel gene expression of catalase. (b) Comparison SOD-2 gene expression between group 1 and group 2. (b′) Comparison of the agarose 1% gel gene expression of SOD-2. All control values were normalized to 1. ^∗∗∗^*p* values < 0.001; AEU: arbitrary expression units.

SOD-2 gene expression demonstrated upregulation in the 1 h (3.012 AEU), 24 h (2.994 AEU), and 72 h (3.010 AEU) telmisartan treatment subgroups compared with the control subgroups (*p* < 0.0006) and demonstrated a decrease in the 7-day (0.5049 AEU) and 14-day (0.5669 AEU) telmisartan subgroups (*p* < 0.0006) (Figure 2(a)).

CAT gene expression demonstrated significant upregulation in the 1 h telmisartan subgroup (1.916 AEU) compared with the control subgroups (*p* < 0.001). No differences were detected in the 72 h subgroup; however, a significant decrease in gene expression was detected in the 24 h (0.771 AEU), 7-day (0.060 AEU), and 14-day (0.334 AEU) telmisartan subgroups compared with the control subgroups (*p* < 0.0006) (Figure 2(b)).

### 4.2. Effect of Telmisartan on Catalase and Superoxide Dismutase Protein Levels

**Figure 3:**
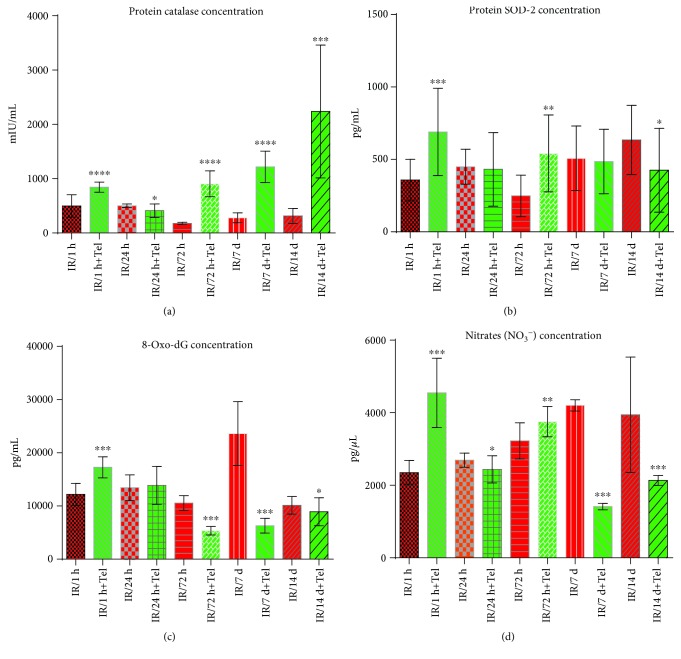
Concentration of enzyme scavengers and oxidative metabolites. Comparison between therapeutic intervention (telmisartan) and control groups: (a) catalase protein concentration; (b) SOD-2 protein concentration; (c) 8-oxo-dG concentration; (d) nitrate concentration. ^∗^*p* ≤ 0.05, ^∗∗^*p* ≤ 0.005, ^∗∗∗^*p* ≤ 0.0005, and ^∗∗∗∗^*p* ≤ 0.00005. mIU/mL: milli-international units per milliliter; pg/mL: picograms per milliliter; pg/*μ*L: picograms per microliter.

CAT protein levels were significantly increased in the 1 h (844.3 mIU/mL), 72 h (905.5 mIU/mL), 7-day (1218 mIU/mL), and 14-day (2239 mIU/mL) telmisartan subgroups compared with the control subgroups (*p* < 0.05); however, these levels were decreased in the 24 h telmisartan subgroup (410.6 mIU/mL) compared with the control subgroups (*p* < 0.05) (Figure 3(a)). In the intragroup comparison of the telmisartan subgroups, a significant decrease in the CAT protein levels was found between the 1 h and 24 h subgroups, although the remaining subgroups showed a significant progressive increase from the 24 h to the 14-day subgroup (*p* < 0.0001) (Table 1).

SOD protein levels showed a significant increase in the 1 h (689.8 pg/mL) and 72 h (540.9 pg/mL) telmisartan subgroups compared with the control subgroups (*p* < 0.05). Furthermore, these levels demonstrated a significant decrease in the 14-day (425.2 pg/mL) telmisartan subgroup compared with the control subgroups (*p* < 0.05); however, no differences were observed between the 24 h and 7-day subgroups (Figure 3(b)). In the intragroup comparison of the telmisartan subgroups, a significant decrease in the levels was found between the 1 h and other subgroups (*p* < 0.001), but no differences were found among the 24 h, 72 h, 7 day, and 14 day subgroups (Table 1).

### 4.3. Effect of Telmisartan on Oxidative Metabolites

8-Oxo-dG concentration was significantly decreased in the 72 h (5,347 pg/mL), 7-day (6,296 pg/mL), and 14-day (8,926 pg/mL) telmisartan subgroups compared with the control subgroups (*p* < 0.001); however, the 24 h subgroup did not show any significant difference. Furthermore, 8-oxo-dG levels were significantly increased in the 1 h telmisartan subgroup (17,271 pg/mL) compared with the control subgroups (*p* < 0.001) (Figure 3(c)). In the intragroup comparison of the telmisartan subgroups, a significant and progressive decrease was observed in 8-oxo-dG levels from the 1 h to the 72 h subgroups (*p* < 0.001); nevertheless, a progressive increase was detected in the 7-day and 14-day subgroups compared with the other subgroups (*p* < 0.0001) (Table 1).

Nitrate concentration was significantly decreased in the 24 h (2,438 pg/*μ*L), 7-day (1,412 pg/*μ*L), and 14-day (2,131 pg/*μ*L) telmisartan subgroups compared with the control subgroups (*p* < 0.005). A significant nitrate concentration increase was detected in the 1 h (4,549 pg/*μ*L) and 72 h (3,754 pg/*μ*L) telmisartan subgroups compared with the control subgroups (*p* < 0.0001 and *p* < 0.0005, respectively) (Figure 3(d)). In the intragroup comparisons of the telmisartan subgroups, three significant peaks of nitrate concentration increase in the 1 h, 72 h, and 14-day (*p* < 0.0001) telmisartan subgroups as well as two significant peaks of decrease in the 24 h and 7-day telmisartan subgroups were observed (*p* < 0.001) (Table 1).

### 4.4. Effect of Telmisartan on Tissue Lesions

**Figure 4:**
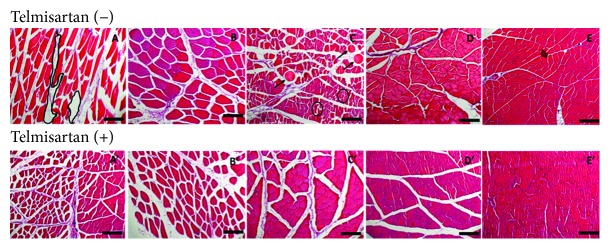
Histological hematoxylin and eosin stain evaluation. (A) I/R 1 h control group: in the delineated black mark, the increase of the interstitial space is observed. (A′) I/R+Telm 1 h group. (B) I/R 24 h control group. (B′) I/R+Telm 24 h group. (C) I/R 72 h control group: the black arrows pinpoint the absence of muscular cells with the delimitation of the stain; the black circles show fragile places at the moment of the cut. (C′) I/R+Telm 72 h group. (D) I/R 7-day control group. (D′) I/R+Telm 7-day group. (E) I/R 14-day control group: its observed reorganization and compaction of the tissue. (E′) I/R+Telm 14-day group: its observed reorganization and compaction of the tissue; inflammatory cells are increasing in comparison with the control group. The black scale bar represents 200 *μ*m. 100x in all pictures.

The lesion assessment (Figure 4) was performed using full-frame counting, fourfold, and stratified techniques. The comparison was done using three variables: lesioned muscle fibers, inflammatory cell infiltrates, and nonlesioned fibers [27]. Tissue damage was defined as a fiber injury characterized by broken or ragged borders, inconsistent texture and color throughout the myocyte, presence of holes, and/or detached nuclei. Uninjured muscular fibers were characterized by well-defined borders, uniform texture and color throughout the myocyte, and absence of holes or ruptures in membranes. Pericellular nuclei or satellite cells could be observed adjacent to uninjured myocytes. Because of the hematoxylin and eosin (H&E) stain, it is not possible to precisely differentiate the type of inflammatory cells, so all the inflammatory cells were counted one by one and evaluated as a whole, using the same technique for each picture, and independently by three different pathologists.

**Figure 5:**
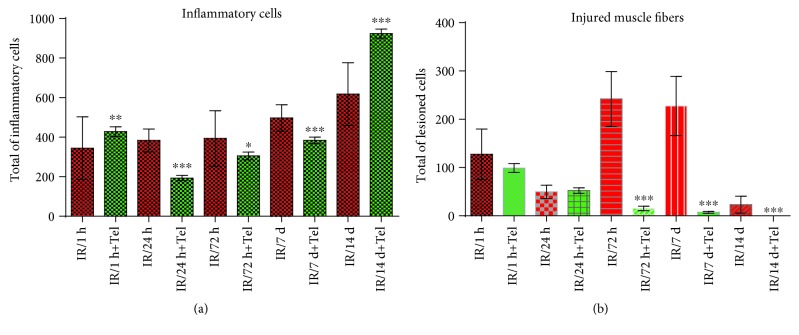
Inflammatory and injured cells. Comparison between therapeutic intervention (telmisartan) and control groups: (a) full count of inflammatory cells; (b) full count of injured cells. ^∗^*p* ≤ 0.05, ^∗∗^*p* ≤ 0.005, and ^∗∗∗^*p* ≤ 0.0005.

The inflammatory cell infiltrate was significantly decreased in the 24 h (193.3 cells), 72 h (305.3 cells), and 7-day (383.8 cells) telmisartan subgroups compared with the control subgroups (*p* < 0.007, *p* < 0.003, and *p* < 0.01, respectively) (Figure 5(a)). Furthermore, the inflammatory cell infiltrate was increased in the 1 h (427.8 cells) and 14-day (923.4 cells) telmisartan subgroups compared with the control subgroups (*p* < 0.0001 and *p* < 0.007, respectively) (Figure 5(a)). In the intragroup comparison of the telmisartan subgroups, a significant decrease was detected in the 24 h subgroup (*p* < 0.001); nevertheless, a significant progressive increase from the 24 h subgroup to the 14-day subgroup was detected (*p* < 0.0001) (Table 1).

The number of injured fibers was decreased in the 72 h (15.5 cells), 7-day (7.4 cells), and 14-day (0 cells) telmisartan subgroups compared with the control subgroups (*p* < 0.0008, *p* < 0.01, and *p* < 0.0001, respectively). No significant differences were detected in the 1 h and 24 h telmisartan subgroups (Figure 5(b)). In the intragroup comparisons of the telmisartan subgroups, a progressive decrease until the 14-day subgroup was detected (*p* < 0.0001) (Table 1).

## 5. Discussion

**Figure 6:**
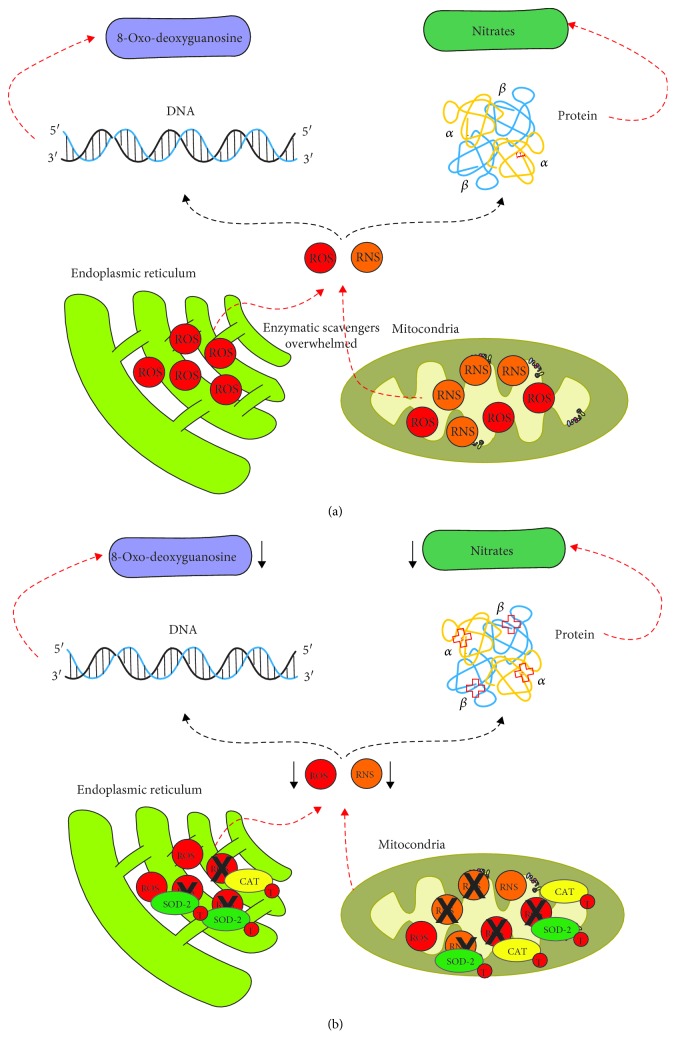
Modulation of oxidative stress during the I/R lesion. (a) Production of OS during the I/R lesion without telmisartan; overproduction of ROS and RNS overpasses the enzymatic scavenger systems, increasing the damage of the components of the cell such as DNA and proteins. (b) Modulation of OS by telmisartan; producing the increase of SOD and CAT that induces reduction of ROS and RNS.

OS has been shown to play an important role in the development and propagation of permanent injury in I/R lesions [1–3]. Under physiological conditions, scavenger systems, including the enzymes such as SOD and CAT, control OS [10, 30–32]. The enzymes are initially responsible for the reduction of excess OS, and their role in signaling redox modulation is crucial. However, during reperfusion, these systems are overwhelmed, and the overproduction of free radicals (ROS and RNS) results in the oxidation of multiple cellular molecules through such proteins (NO_3_^−^) and DNA (8-oxo-dG) (Figure 6). Telmisartan has been described as a modulator of some oxidative and antioxidant biomarkers, but its mechanism of action is not well understood [15, 18, 20, 21, 33–39]. In the present study, we found that telmisartan produced changes in the SOD-2 and CAT gene expression but to a different degree and time-course regarding reperfusion.

CAT expression modulation has been described as an adaptive response for cell survival after OS injury; furthermore, telmisartan treatment after OS injury has been shown to increase CAT activity and concentration [39–41]. We observed an increase in CAT gene expression and protein concentration only in the 1 h subgroup without any changes in the concentration of oxidative biomarkers and extent of tissue injury. Nevertheless, CAT protein levels decreased in the 24 h subgroup and subsequently persistently increased in the 72 h, 7-day, and 14-day subgroups; this was associated with a decrease in the extent of injured tissues and expression of oxidative markers.

CAT gene expression is a complex process; this is positively regulated by the presence of PPAR-*γ* through a response element at −12 kb from the transcription initiation site of the human catalase gene. Excess reactive oxygen and nitrogen species can induce a negative CAT and PPAR-*γ* gene expression [40, 42–46], reducing CAT and PPAR-*γ* protein levels in the affected tissues. In addition, during the reparation process, the signaling pathways mediated by TGF-*β* and TNF-*α* were activated throughout the inflammatory processes, consequently downregulating the CAT gene expression [40, 42–46]. Telmisartan directly increases PPAR-*γ* gene and protein expression, consecutively increasing the CAT gene expression [39, 41].

The behavior of the CAT gene expression during the first hour reflected a preconditioning effect, which produces an overexpression of CAT, increases the protein levels, and prepares the cells to tolerate oxidative stress. However, at 24 h of reperfusion, we can see a significant decrease in the CAT gene expression due to the overproduction of ROS and RNS, which surpasses the preconditioning effect caused by telmisartan. This negative feedback in the CAT gene expression is observed in all body cells due to the fact that ROS and RNS are released in the body; however, telmisartan could have an effect in the nonaffected cells, which are overexpressing CAT; thus, we can see a normalization on the levels of CAT gene expression and an increase in the levels of CAT protein in serum at 72 h. As a consequence to I/R in locally affected tissues, we see a decrease of the CAT gene expression at 7 and 14 days accompanied with an increase of CAT protein in serum. To explain this behavior, we could take into account the downregulation induced by the activation of the inflammatory and reparation mechanisms (TGF-*β* and TNF-*α*) in the affected tissues at 72 h, which decrease gene expression. The increase of the CAT protein is related to the continuous effect of telmisartan on the nonaffected cells. These changes correlate with the tissue reparation, which starts at 72 h and is completely established at 14 days (histological images). The direct and indirect effects of telmisartan in the immune system were beneficial to the early recovery of the damaged cells. The histological stains with a higher immune cell count demonstrated a better recruitment of inflammatory cells in the tissue affected by I/R and displayed a faster recovery of the tissue compared with those not treated with telmisartan.

SOD-2 is the first enzymatic scavenger after an OS perturbation, and its expression is upregulated when an inflammatory process is coactivated [47–52]. In our study, SOD-2 gene expression (in the 1 h, 24 h, and 72 h subgroups) and protein levels (in the 1 h and 72 h subgroups) were increased; however, this high gene and protein expression apparently had no impact on the extent of tissue injury in the 1 h subgroup (Figure 5(b)). Nevertheless, among the telmisartan subgroups, we observed a decrease in gene expression in the 7- and 14-day subgroups, but not in the protein levels in the 24 h and 14-day subgroups. We did not measure enzyme activity; however, we propose that telmisartan may increase CAT and SOD bioavailability because we detected an increase in protein levels without gene expression increase after treatment with it.

The variability in NO synthase explains the behavior of the levels of nitrates during the experiment, due to the fact that it is modulated by multiple mechanisms, local and systemic [53–55]. There are differences in these regulation mechanisms for the different isoforms of this enzyme (endothelial, neuronal, mitochondrial, and inducible) [53–58].

Telmisartan and other ARB increase the expression and the activity of eNOS in endothelial cells, giving protection in circumstances of stress [53, 55, 59]. However, in the presence of severe hypoxia with an excessive production of ROS and RNS, eNOS will induce a rearrangement of these enzymes in the cellular membrane and a malfunctioning that will affect the production of NO [35, 53, 54, 56, 57, 60].

Great quantities of ROS cause a systemic effect that decreases the expression and activity of SOD, which coincides with the decrease of nitrates we observed during the first 24 hours and is related with the inactivity of eNOS. We consider that once the activity of endothelial eNOS and ROS is regulated through other modulation mechanisms such as those mediated by the immune system, there is a decrease in the concentration of systemic nitrates, which is what we would observe at 72 hours and 14 days.

Telmisartan may decrease oxidative biomarker levels and improve injury recovery through OS and inflammatory response modulation in the local tissue. In our study, telmisartan's effect on injury attenuation and OS product reduction was present between 24 h and 14 days. Analyzing the lesion extent and progress in nontreated rats from the start (1 h) to the time when recovery of the lesion (14 days) is observed, we conclude that the progress of the I/R lesion is divided into two periods. The first is damage limitation that can occur until 7 days after the start of the reperfusion. The second is the start of the reparation process that can be underhanded since the first 72 h after the start of the reperfusion. The repair mechanism activation involves many regulatory steps that are influenced by local and systemic responses that exceed the scope of this study. Nevertheless, these two processes are probably coactivated by several gene regulation and signaling mechanisms. One of the most important check points should be OS modulation because we demonstrated that enzyme system modulation improves the damage limitation and the recovery process. We also evaluated the presence of immune modulation by telmisartan [19, 20, 33, 37, 61, 62], apparently increasing the recovery effect on the muscle skeletal cells. Telmisartan affected the inflammatory response by causing a continuous increase in inflammatory cell infiltration, but at the same time, tissue damage improvement in the muscle skeletal cells was demonstrated for the first time. Apparently, the influence of telmisartan on gene expression within the first 72 h and the increase in the bioavailability of enzyme systems that modulate OS help in the upregulation of the reparatory immune cells, besides overactivation of the damage limitation of the immune system. However, because no specific biomarkers of inflammatory response and histological markers exist, no definitive conclusions can be drawn.

## 6. Conclusions

**Figure 7:**
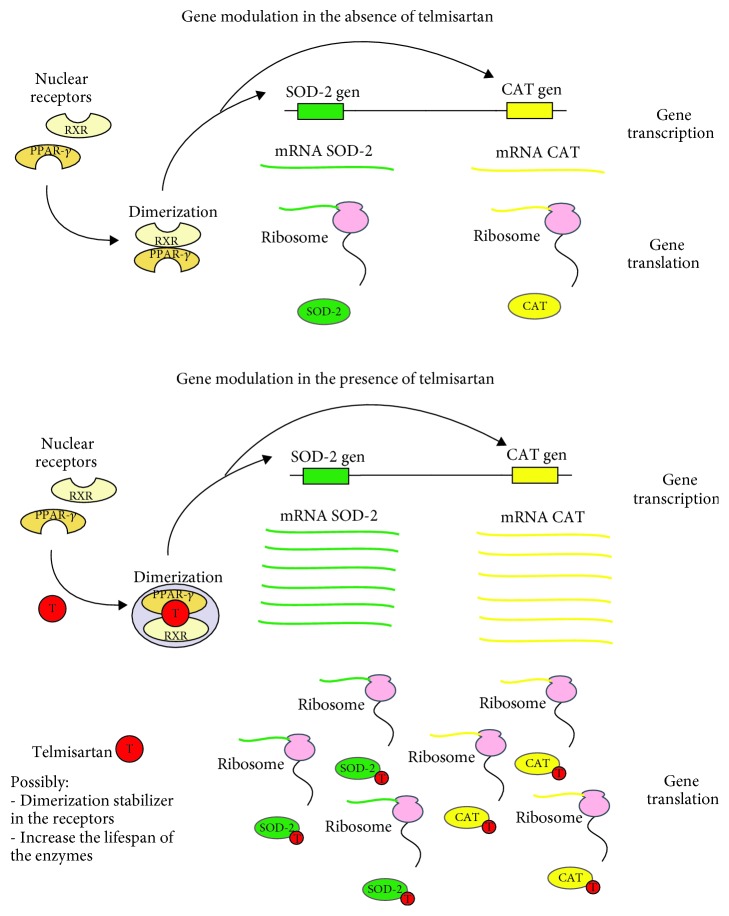
Telmisartan's possible mechanisms of action.

Telmisartan treatment results in the upregulation of the enzyme scavengers SOD and CAT during an I/R lesion formation, possibly not only through gene expression induction but also through prolongation of the lifespan of the enzymes (Figure 7). Modulation of gene expression of SOD and CAT levels contributes to the recovery as well as to a decrease in the damage during the late phase of the I/R lesion.

## Figures and Tables

**Table 1:** Correlation of all results.

Groups	1 hour		24 hours		72 hours		7 days		14 days	
Biomarker	C	T	C	T	C	T	C	T	C	T
SOD-2 gen (AEU)	1	3.012 (SD 0.264)	1	2.994 (SD 0.187)	1	3.01 (SD 0.263)	1	0.50 (SD 0.023)	1	0.566 (SD 0.023)
SOD-2 protein (pg/mL)	357.6 (SD 142.9)	689.8 (SD 301.4)	449.9 (SD 120.3)	431.4 (SD 254.8)	248.2 (SD 143.2)	540.9 (SD 265.7)	507.4 (SD 22.8)	486.1 (SD 22.6)	634.2 (SD 239.3)	425.2 (SD 289)
CAT gen (AEU)	1	1.916 (SD 0.184)	1	0.771 (SD 0.056)	1	1.036 (SD 0.083)	1	0.060 (SD 0.002)	1	0.334 (SD 0.052)
CAT protein (mIU/mL)	498.4 (SD 205.9)	844.3 (SD 92.43)	501.6 (SD 34.35)	410.6 (SD 123.1)	177.5 (SD 20.15)	905.5 (SD 238.4)	279.9 (SD 92.5)	1,218 (SD 290.1)	315 (SD 135.7)	2239 (SD 1,223)
8-Oxo-dG (pg/mL)	12,168 (SD 2,091)	17,271 (SD 1,981)	13,442 (SD 2,411)	13,877 (SD 3,559)	10,552 (SD 1396)	5,347 (SD 807.2)	23,609 (SD 6,002)	6,296 (SD 1,394)	10,125 (SD 1,653)	8,926 (SD 2,630)
NO_3_^−^ (pg/*μ*L)	2,346 (SD 334.3)	4,549 (SD 954.8)	2,694 (SD 194.7)	2,438 (SD 376.2)	3,224 (SD 497)	3,754 (SD 418.6)	4,202 (SD 154.1)	1,412 (SD 90.72)	3,943 (SD 1,587)	2,133 (SD 133.9)
Inflammatory cells (full count)	344.8 (SD 158.1)	427.8 (SD 24.63)	383.5 (SD 58.09)	193.3 (SD 13.44)	393.4 (SD 140.4)	305.3 (SD 19.67)	497.2 (SD 66.7)	383.8 (SD 16.54)	618 (SD 158.4)	923.4 (SD 22.58)
Injured cells (full count)	127.5 (SD 52.45)	98.85 (SD 9.17)	49.75 (SD 13.89)	52.50 (SD 5.73)	242 (SD 56.78)	15.50 (SD 4.5)	227.4 (SD 61.3)	7.4 (SD 1.93)	23.25 (SD 17.42)	0

All values are in mean and with confidence interval of 95%. C: control groups; T: telmisartan groups; SD: standard deviation; AEU: arbitrary expression units; pg/mL: picograms per milliliter; pg/*μ*L: picograms per microliter; mIU/mL: milli-international units per milliliter.

## Data Availability

We guarantee the veracity of the data shown here, and if you require us, we can provide the databases of each one of the experiments and the authorization document of the ethics and research committee.

## References

[1] Kalogeris T., Baines C. P., Krenz M., Korthuis R. J. (2016). Ischemia/reperfusion. *Comprehensive Physiology*.

[2] Rodríguez-Lara S. Q., García-Benavides L., Miranda-Díaz A. G. (2018). The renin-angiotensin-aldosterone system as a therapeutic target in late injury caused by ischemia-reperfusion. *International Journal of Endocrinology*.

[3] Rodríguez-Lara S. Q., Cardona-Muñoz E. G., Ramírez-Lizardo E. J. (2016). Alternative interventions to prevent oxidative damage following ischemia/reperfusion. *Oxidative Medicine and Cellular Longevity*.

[4] Kalogeris T., Baines C. P., Krenz M., Korthuis R. J. (2012). Cell biology of ischemia/reperfusion injury. *International Review of Cell and Molecular Biology*.

[5] Sanchis-Gomar F., Perez-Quilis C., Leischik R., Lucia A. (2016). Epidemiology of coronary heart disease and acute coronary syndrome. *Annals of Translational Medicine*.

[6] Ziaeian B., Fonarow G. C. (2016). Epidemiology and aetiology of heart failure. *Nature Reviews Cardiology*.

[7] Smajlović D. (2015). Strokes in young adults: epidemiology and prevention. *Vascular Health and Risk Management*.

[8] Powers W. J., Rabinstein A. A., Ackerson T. (2018). 2018 guidelines for the early management of patients with acute ischemic stroke: a guideline for healthcare professionals from the American Heart Association/American Stroke Association. *Stroke*.

[9] Hausenloy D. J., Yellon D. M. (2013). Myocardial ischemia-reperfusion injury: a neglected therapeutic target. *The Journal of Clinical Investigation*.

[10] Birben E., Sahiner U. M., Sackesen C., Erzurum S., Kalayci O. (2012). Oxidative stress and antioxidant defense. *World Allergy Organization Journal*.

[11] Penna C., Mancardi D., Raimondo S., Geuna S., Pagliaro P. (2008). The paradigm of postconditioning to protect the heart. *Journal of Cellular and Molecular Medicine*.

[12] Lee S. Y., Kang K. N., Kang J. H. (2017). Pharmacokinetics of a telmisartan, amlodipine and hydrochlorothiazide fixed-dose combination: a replicate crossover study in healthy Korean male subjects. *Tropical Journal of Pharmaceutical Research*.

[13] Patel P. (2017). Telmisartan: clinical evidence across the cardiovascular and renal disease continuum. *Drugs & Therapy Perspectives*.

[14] Fan Y., Zhang D., Xiang D. (2016). Delayed protective effect of telmisartan on lung ischemia/reperfusion injury in valve replacement operations. *Experimental and Therapeutic Medicine*.

[15] Eslami H., Sharifi A. M., Rahimi H., Rahati M. (2014). Protective effect of telmisartan against oxidative damage induced by high glucose in neuronal PC12 cell. *Neuroscience Letters*.

[16] Ozeki K., Tanida S., Morimoto C. (2013). Telmisartan inhibits cell proliferation by blocking nuclear translocation of ProHB-EGF C-terminal fragment in colon cancer cells. *PLoS One*.

[17] Takeuchi K., Yamamoto K., Ohishi M. (2013). Telmisartan modulates mitochondrial function in vascular smooth muscle cells. *Hypertension Research*.

[18] Fujita H., Fujishima H., Morii T. (2012). Modulation of renal superoxide dismutase by telmisartan therapy in C57BL/6-*Ins2^Akita^* diabetic mice. *Hypertension Research*.

[19] Pang T., Benicky J., Wang J., Orecna M., Sanchez-Lemus E., Saavedra J. M. (2012). Telmisartan ameliorates lipopolysaccharide-induced innate immune response through *peroxisome proliferator-activated receptor-γ* activation in human monocytes. *Journal of Hypertension*.

[20] Pang T., Wang J., Benicky J., Sanchez-Lemus E., Saavedra J. M. (2012). Telmisartan directly ameliorates the neuronal inflammatory response to IL-1*β* partly through the JNK/c-Jun and NADPH oxidase pathways. *Journal of Neuroinflammation*.

[21] Fujita H., Sakamoto T., Komatsu K. (2011). Reduction of circulating superoxide dismutase activity in type 2 diabetic patients with microalbuminuria and its modulation by telmisartan therapy. *Hypertension Research*.

[22] Kumtepe Y., Odabasoglu F., Karaca M. (2010). Protective effects of telmisartan on ischemia/reperfusion injury of rat ovary: biochemical and histopathologic evaluation. *Fertility and Sterility*.

[23] Lehrke M., Lazar M. A. (2005). The many faces of PPAR*γ*. *Cell*.

[24] Camps J., García-Heredia A., Rull A. (2012). PPARs in regulation of paraoxonases: control of oxidative stress and inflammation pathways. *PPAR Research*.

[25] UU E National Research Council (2001). *Guide for the Care and Use of Laboratory Animals*.

[26] Freeman W. M., Walker S. J., Vrana K. E. (1999). Quantitative RT-PCR: pitfalls and potential. *BioTechniques*.

[27] McCormack M. C., Kwon E., Eberlin K. R. (2008). Development of reproducible histologic injury severity scores: skeletal muscle reperfusion injury. *Surgery*.

[28] Hu C., Muller-Karger F. E., Zepp R. G. (2002). Absorbance, absorption coefficient, and apparent quantum yield: a comment on common ambiguity in the use of these optical concepts. *Limnology and Oceanography*.

[29] VanGuilder H. D., Vrana K. E., Freeman W. M. (2008). Twenty-five years of quantitative PCR for gene expression analysis. *BioTechniques*.

[30] Demidchik V. (2015). Mechanisms of oxidative stress in plants: from classical chemistry to cell biology. *Environmental and Experimental Botany*.

[31] Daiber A., Mader M., Stamm P. (2013). Oxidative stress and vascular function. *Cell Membranes and Free Radical Research*.

[32] Jones D. P. (2006). Redefining oxidative stress. *Antioxidants & Redox Signaling*.

[33] Kelleni M. T., Ibrahim S. A., Abdelrahman A. M. (2016). Effect of captopril and telmisartan on methotrexate-induced hepatotoxicity in rats: impact of oxidative stress, inflammation and apoptosis. *Toxicology Mechanisms and Methods*.

[34] Dessì M., Piras A., Madeddu C. (2011). Long-term protective effects of the angiotensin receptor blocker telmisartan on epirubicin-induced inflammation, oxidative stress and myocardial dysfunction. *Experimental and Therapeutic Medicine*.

[35] Knorr M., Hausding M., Kröller-Schuhmacher S. (2011). Nitroglycerin-induced endothelial dysfunction and tolerance involve adverse phosphorylation and S-glutathionylation of endothelial nitric oxide synthase: beneficial effects of therapy with the AT1 receptor blocker telmisartan. *Arteriosclerosis, Thrombosis, and Vascular Biology*.

[36] Washida K., Ihara M., Nishio K. (2010). Nonhypotensive dose of telmisartan attenuates cognitive impairment partially due to peroxisome proliferator-activated Receptor-*γ* activation in mice with chronic cerebral hypoperfusion. *Stroke*.

[37] Cianchetti S., Del Fiorentino A., Colognato R., Di Stefano R., Franzoni F., Pedrinelli R. (2008). Anti-inflammatory and anti-oxidant properties of telmisartan in cultured human umbilical vein endothelial cells. *Atherosclerosis*.

[38] Matsui T., Yamagishi S., Ueda S. (2007). Telmisartan, an angiotensin II type 1 receptor blocker, inhibits advanced glycation end-product (AGE)-induced monocyte chemoattractant protein-1 expression in mesangial cells through downregulation of receptor for AGEs via peroxisome proliferator-activated receptor-*γ* activation. *Journal of International Medical Research*.

[39] Sugiyama H., Kobayashi M., Wang D.-H. (2005). Telmisartan inhibits both oxidative stress and renal fibrosis after unilateral ureteral obstruction in acatalasemic mice. *Nephrology Dialysis Transplantation*.

[40] Goyal M. M., Basak A. (2010). Human catalase: looking for complete identity. *Protein & Cell*.

[41] Iqbal M., Dubey K., Anwer T., Ashish A., Pillai K. K. (2008). Protective effects of telmisartan against acute doxorubicin-induced cardiotoxicity in rats. *Pharmacological Reports*.

[42] Glorieux C., Zamocky M., Sandoval J. M., Verrax J., Calderon P. B. (2015). Regulation of catalase expression in healthy and cancerous cells. *Free Radical Biology & Medicine*.

[43] Kodydková J., Vávrová L., Kocík M., Zak A. (2014). Human catalase, its polymorphisms, regulation and changes of its activity in different diseases. *Folia Biologica*.

[44] Okuno Y., Matsuda M., Miyata Y. (2010). Human catalase gene is regulated by peroxisome proliferator activated receptor-gamma through a response element distinct from that of mouse. *Endocrine Journal*.

[45] Okuno Y., Matsuda M., Kobayashi H. (2008). Adipose expression of catalase is regulated via a novel remote PPAR*γ*-responsive region. *Biochemical and Biophysical Research Communications*.

[46] Girnun G. D., Domann F. E., Moore S. A., Robbins M. E. C. (2002). Identification of a functional peroxisome proliferator-activated receptor response element in the rat catalase promoter. *Molecular Endocrinology*.

[47] Nolly M. B., Caldiz C. I., Yeves A. M. (2014). The signaling pathway for aldosterone-induced mitochondrial production of superoxide anion in the myocardium. *Journal of Molecular and Cellular Cardiology*.

[48] Tran T. P., Tu H., Pipinos I. I., Muelleman R. L., Albadawi H., Li Y. L. (2011). Tourniquet-induced acute ischemia–reperfusion injury in mouse skeletal muscles: involvement of superoxide. *European Journal of Pharmacology*.

[49] Grivennikova V. G., Vinogradov A. D. (2006). Generation of superoxide by the mitochondrial complex I. *Biochimica et Biophysica Acta (BBA) - Bioenergetics*.

[50] Estevez A. G., Jordan J. (2002). Nitric oxide and superoxide, a deadly cocktail. *Annals of the New York Academy of Sciences*.

[51] Messner K. R., Imlay J. A. (2002). Mechanism of superoxide and hydrogen peroxide formation by fumarate reductase, succinate dehydrogenase, and aspartate oxidase. *Journal of Biological Chemistry*.

[52] Bhakdi S., Martin E. (1991). Superoxide generation by human neutrophils induced by low doses of Escherichia coli hemolysin. *Infection and Immunity*.

[53] Förstermann U., Sessa W. C. (2012). Nitric oxide synthases: regulation and function. *European Heart Journal*.

[54] Förstermann U., Li H. (2011). Therapeutic effect of enhancing endothelial nitric oxide synthase (eNOS) expression and preventing eNOS uncoupling. *British Journal of Pharmacology*.

[55] Kleinert H., Art J., Pautz A. (2010). Regulation of the expression of inducible nitric oxide synthase. *Nitric Oxide*.

[56] Förstermann U., Xia N., Li H. (2017). Roles of vascular oxidative stress and nitric oxide in the pathogenesis of atherosclerosis. *Circulation Research*.

[57] Davidson S. M., Duchen M. R. (2007). Endothelial mitochondria: contributing to vascular function and disease. *Circulation Research*.

[58] Ghafourifar P., Cadenas E. (2005). Mitochondrial nitric oxide synthase. *Trends in Pharmacological Sciences*.

[59] Sampaio W. O., Souza dos Santos R. A., Faria-Silva R., da Mata Machado L. T., Schiffrin E. L., Touyz R. M. (2007). Angiotensin-(1-7) through receptor Mas mediates endothelial nitric oxide synthase activation via Akt-dependent pathways. *Hypertension*.

[60] Thomas D. D., Liu X., Kantrow S. P., Lancaster J. R. (2001). The biological lifetime of nitric oxide: implications for the perivascular dynamics of NO and O_2_. *Proceedings of the National Academy of Sciences of the United States of America*.

[61] Nakano A., Hattori Y., Aoki C., Jojima T., Kasai K. (2009). Telmisartan inhibits cytokine-induced nuclear factor-*κ*B activation independently of the peroxisome proliferator-activated receptor-*γ*. *Hypertension Research*.

[62] Tian Q., Miyazaki R., Ichiki T. (2009). Inhibition of tumor necrosis factor-*α*–induced interleukin-6 expression by telmisartan through cross-talk of peroxisome proliferator-activated receptor-*γ* with nuclear factor *κ*B and CCAAT/enhancer-binding protein-*β*. *Hypertension*.

